# The association analysis of TLR2 and TLR4 gene with tuberculosis in the Tibetan Chinese population

**DOI:** 10.18632/oncotarget.22996

**Published:** 2017-12-06

**Authors:** Xin Xue, Yi Qiu, Dong Jiang, Tianbo Jin, Mengdan Yan, Xikang Zhu, Yonglie Chu

**Affiliations:** ^1^ Department of Pathogen Biology and Immunology, Xi’an Jiaotong University Health Science Center, Xi’an 710061, China; ^2^ The Fourth Internal Medicine, Xi’an Chest Hospital, Xi’an TB and Thoracic Tumor Hospital, Xi’an 710100, China; ^3^ The Second Internal Medicine, Xi’an Chest Hospital, Xi’an TB and Thoracic Tumor Hospital, Xi’an 710100, China; ^4^ Inner Mongolia Medical University, Hohhot 010010, China; ^5^ Key Laboratory of Resource Biology and Biotechnology in Western China (Northwest University), Ministry of Education, School of Life Sciences, Northwest University, Xi’an 710069, China; ^6^ School of Medicine, Xizang Minzu University, Xianyang 712082, China

**Keywords:** PTB, SNP, case-control study, Tibetan Chinese population

## Abstract

**Background:**

The present study was undertaken to explore the relationship of Toll-like receptor (TLR) 2, TLR4 genes polymorphisms with Pulmonary tuberculosis (PTB) risk in a sample of Chinese population.

**Methods:**

For this study, we recruited 467 subjects with PTB and 504 healthy subjects from a Tibetan population living in near or in Xi’an, China. Association analyses of single-nucleotide polymorphisms (SNPs) in *TLR2 and TLR4* were performed with SPSS Statistics (version 17.0), SNPStats, Haploview (version 4.2), and SHEsis software.

**Results:**

The research results that is association analysis of pulmonary tuberculosis show there are two increased-risk SNPs (rs7696323, OR=1.32, 95%CI =1.08-1.62, *P*= 0.007; rs12377632, OR=1.30, 95%CI =1.09-1.55, *P*= 0.004) and three decreased-risk SNPs (rs3804099, OR=0.64, 95%CI =0.52-0.79, *P*= 1.9510^−5^; rs3804100, OR=0.67, 95%CI =0.54-0.82, *P*= 0.0001; rs11536889, OR=0.54, 95%CI =0.42-0.69, *P*= 9.1410^−7^).

**Conclusions:**

We found that two SNPs are associated with increased PTB risk and three SNPs decreased PTB risk in the Chinese Tibetan population. Our findings demonstrate an association between *TLR2* and *TLR4* polymorphisms and PTB.

## INTRODUCTION

Pulmonary tuberculosis (PTB) is caused by *Mycobacterium tuberculosis* (*M. tuberculosis*) and is one of the leading causes of death worldwide. Since 1980, the PTB incidence and mortality rate have increased rapidly [1]. There are more than 9 million people with PTB, and more than 1.7 million people have died of PTB [2]. The incidence of PTB in Asian countries accounts for 60% of the worldwide total. The number of PTB patients in China is the second largest in the world, and China is ranked on a list of 22 high-burden countries. Although *M. tuberculosis* has infected almost one-third of the population worldwide, only 10% of patients produce corresponding clinical symptoms during their lifetime [3], illustrating that additional factors may influence the incidence of disease among different individuals.

Previous studies have identified genes that confer disease susceptibility by regulating the immune response [4, 5]. A twin study found that PTB concordance in identical twins is2-fold higher than that in non-identical twins [6]. Thus, we expect that identification of host genetic factors for PTB susceptibility might play a key role in PTB control worldwide. Genetic research has provided insight into tuberculosis, including the pathological and cytological bases of PTB. Several genes that influence PTB risk have been identified, including *VDR*, *MBL*, *TLR*, and *P2×7* et al. [7–9].

Among them, the polymorphism of TLR2 and TLR4 genes were considered to be the most closely related Toll-like receptor members of immunity and inflammation. The cooperation between TLR2 and TLR4 dependent signaling plays a crucial role in macrophage apoptosis triggered by PTB [10].

However, the association analysis between TLRs (especially TLR2 and TLR4) and PTB risk have been reported, few geneticists and cliniciansassess the association between TLR2, TLR4 and TB risk in the Tibetan Chinese population. By applying the case-control study method in epidemiology, to evaluate the association between common TLR2, TLR4 single nucleotide polymorphisms (SNPs) and the susceptibility of PTB in an Han Chinese population. We selected 4 tag SNPs of TLR2, 5 tag SNPs of TLR4 to perform a comprehensive association analysis with TB. Establishing our analysis to the risk of PTB loci, some evidences are provided to share hereditary susceptibility between PTB and three genes at 9 new loci in Chinese people.

## RESULTS

Demographic and clinical features of the PTB and the control group are shown in Tables 1 and 2 showed allele frequencies and odd ratio estimates among the subjects in our case-control study. In Hardy-Weinberg balance verification, HWE *P* value is the boundary value of the level of the candidate gene, thus rs1898830 and rs10759932 were excluded. Determination of other seven SNPs in the control group show that they conform to the Weinberg Hardy equilibrium law HWE. There are two increased-risk SNPs (rs7696323, OR=1.32, 95%CI =1.08-1.62, *P*= 0.007; rs12377632, OR=1.30, 95%CI =1.09-1.55, *P*= 0.004) and three decreased-risk SNPs (rs3804099, OR=0.64, 95%CI =0.52-0.79, *P*= 1.95^*^10^−5^; rs3804100, OR=0.67, 95%CI =0.54-0.82, *P*= 0.0001; rs11536889, OR=0.54, 95%CI =0.42-0.69, *P*= 9.14^*^10^−7^).

**Table 1:** Characteristics of PTB patients and control participants

Variable	Case	Control	P
Total	467	503	
Age(Mean±SD)	50.67±7.80	50.34±7.74	0.508
Gender			0.947
Female	287	308	
Male	180	195	

p ≤ 0.05 indicates statistical significance.

**Table 2:** Allele frequencies in cases and controls and odds ratio estimates for tuberculosis

SNP	Chromosome	Position	Band	Alleles A/B	Major allelic frequency		Gene(s)	HWE *P* value	ORs	95% CI		*P* value
					Case	control						
rs7696323	4	154605745	4q31.3	T/C	0.281	0.719	TLR2	0.704	1.32	1.08	1.62	0.007
rs1898830	4	154608453	4q31.3	G/A	0.469	0.531	TLR2	0.031	1.05	0.88	1.26	0.609
rs3804099	4	154624656	4q31.3	C/T	0.226	0.774	TLR2	0.534	0.64	0.52	0.79	1.95E-05^*^
rs3804100	4	154625409	4q31.3	C/T	0.208	0.792	TLR2	0.912	0.67	0.54	0.82	0.0001^*^
rs10759932	9	120465144	9q33.1	C/T	0.378	0.622	TLR4	0.001	1.37	1.13	1.67	0.001
rs11536878	9	120471553	9q33.1	A/C	0.111	0.889	TLR4	0.211	0.88	0.67	1.17	0.383
rs12377632	9	120472730	9q33.1	T/C	0.489	0.511	TLR4	1.000	1.30	1.09	1.55	0.004
rs11536889	9	120478131	9q33.1	C/G	0.121	0.879	TLR4	0.337	0.54	0.42	0.69	9.14E-07^*^
rs7873784	9	120478936	9q33.1	C/G	0.088	0.912	TLR4	1.000	0.96	0.70	1.31	0.778

*SNP* single nucleotide polymorphism, *OR* odds ratio, 95 % *CI* 95 % confidence interval, *HWE* Hardy–Weinberg equilibrium*p* ≤ 0.05 indicates statistical significance.

^*^p value ≤ 0.05 indicates Bonfferoni correction statistical significance.

According to the genetic model analyses (Table 3), the T/T genotype ofrs6687758 increased 2.08-fold risk in the recessive model (*P*< 0.05), the *T/C-C/C* genotype in the dominant model and the C/C genotype in the recessive model of rs3804099 decreased 0.63-fold and 0.43-fold PTB risk (*P*< 0.05), the minor C allele of rs3804100 in the recessive model decreased 0.50-fold risk and the *T/C-C/C* genotype in the dominant model decreased 0.63-fold (*P*< 0.05), the *T/C-C/C* genotype in the dominant model of rs10759932 and the *C/T-T/T* genotype in the dominant model of rs12377632 increased risk of TB based on analysis (The former OR=1.57, 95 % CI=1.21-2.04, *P*=0.0008. The latter OR=1.56, 95 % CI=1.18-2.07, *P*=0.002), the *G/C-C/C* genotype in the dominant model and the *C/C* genotype in the recessive model of rs11536889decreased 0.50-fold and 0.48-fold TB risk (*P*< 0.05).

**Table 3:** Logistic regression analysis of the association between the single-nucleotide polymorphisms and PTB

SNP ID	Model	Genotype	OR	95% CI		*P* value
rs7696323	dominant	C/C	1.00			0.063
		C/T-T/T	1.27	0.99	1.64	
	recessive	C/C-C/T	1.00			0.005
		T/T	2.08	1.24	3.47	
	genotype	T/T	2.19	1.30	3.71	0.003
		C/T	1.15	0.88	1.50	0.305
		C/C	1.00			
rs1898830	dominant	A/A	1.00			0.360
		A/G-G/G	0.88	0.66	1.16	
	recessive	A/A-A/G	1.00			0.056
		G/G	1.36	0.99	1.85	
	genotype	G/G	1.16	0.80	1.67	0.4363
		A/G	0.78	0.58	1.05	0.1057
		A/A	1.00			
rs3804099	dominant	A/A	1.00			0.0003
		T/C-C/C	0.63	0.48	0.81	
	recessive	T/T-T/C	1.00			0.001
		C/C	0.43	0.26	0.72	
	genotype	C/C	0.37	0.22	0.62	0.0002
		T/C	0.69	0.53	0.90	0.006
		T/T	1.00			
rs3804100	dominant	T/T	1.00			0.0004
		T/C-C/C	0.63	0.49	0.82	
	recessive	T/T-T/C	1.00			0.017
		C/C	0.50	0.29	0.89	
	genotype	C/C	0.43	0.24	0.76	0.004
		T/C	0.67	0.51	0.87	0.003
		T/T	1.00			
rs10759932	dominant	T/T	1.00			0.0007
		T/C-C/C	1.57	1.21	2.04	
	recessive	T/T-T/C	1.00			0.173
		C/C	1.30	0.89	1.88	
	genotype	T/T	1.59	1.07	2.37	0.021
		T/C	1.56	1.18	2.07	0.002
		C/C	1.00			
rs11536878	dominant	C/C	1.00			0.257
		C/A-A/A	0.84	0.62	1.14	
	recessive	C/C-C/A	1.00			0.453
		A/A	1.63	0.46	5.80	
	genotype	A/A	1.56	0.44	5.56	0.495
		C/A	0.81	0.60	1.11	0.193
		C/C	1.00			
rs12377632	dominant	C/C	1.00			0.002
		C/T-T/T	1.56	1.18	2.07	
	recessive	C/C-C/T		1.00		0.124
		T/T	1.28	0.93	1.76	
	genotype	T/T	1.68	1.16	2.44	0.006
		C/T	1.52	1.13	2.04	0.006
		C/C	1.00			
rs11536889	dominant	G/G	1.00			1.77E-06
		G/C-C/C	0.50	0.37	0.66	
	recessive	G/G-G/C	1.00			0.046
		C/C	0.48	0.23	0.99	
		C/C	0.40	0.19	0.83	0.0141
		G/C	0.51	0.38	0.69	1.24E-05
		G/G	1.00			
rs7873784	dominant	G/G	1.00			0.743
		G/C-C/C	0.95	0.68	1.32	
	recessive	G/G-G/C	1.00			0.916
		C/C	1.08	0.27	4.33	
		C/C	1.07	0.27	4.30	0.928
		G/C	0.94	0.67	1.32	0.722
		G/G	1.00			

*SNP* single nucleotide polymorphism, *OR* odds ratio, 95 % *CI* 95 % confidence interval.

*p* ≤ 0.05 indicates statistical significance.

*p* value were calculated using two-sided Chi-squared test.

**Figure 1:**
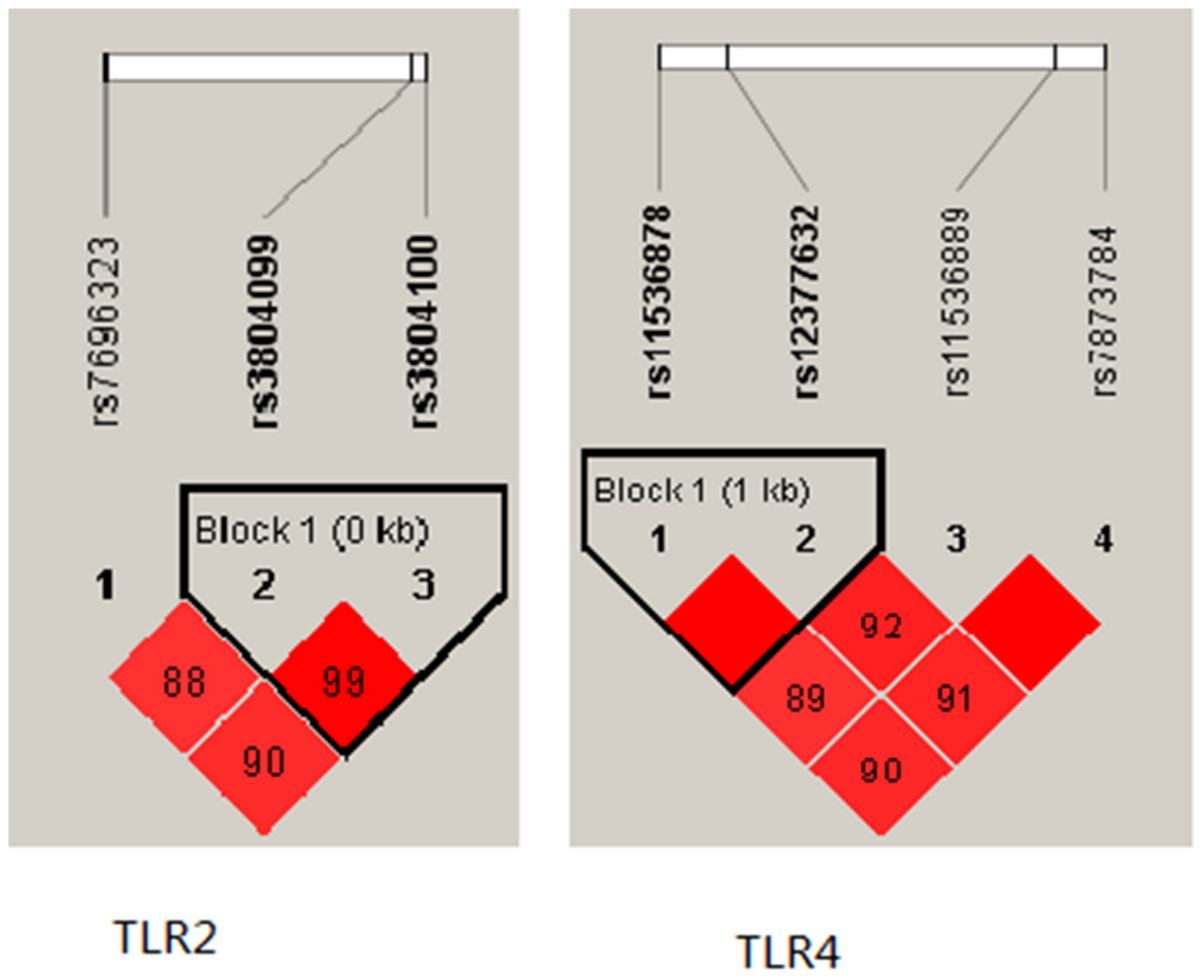
Linkage disequilibrium plot of TLR2 and TLR4SNPs

Finally, a haplotype-based association study was performed to show the association betweenTLR2 haplotype and risk of PTB (Figure 1). We found that the candidate SNPs (rs3804099 and rs3804100) in the TLR2 gene showed strong linkage. We analysed the haplotype association with PTB, the results in Table 4.

**Table 4:** Haplotype frequencies and their associations with PTB risk

Gene	SNPs	Haplotype	OR	95% CI		P
TLR2	rs3804099|rs3804100	CC	0.67	0.54	0.82	0.0002
		CT	0.58	0.31	1.05	0.073
		TT	1.56	1.27	1.91	2.11E-05
TLR4	rs11536878|rs12377632	AT	0.88	0.66	1.17	0.3757
		CT	1.45	1.19	1.76	0.0002
		CC	0.76	0.63	0.91	0.004

## DISCUSSION

The research results that is association analysis of pulmonary tuberculosis show there are two increased-risk SNPs (rs7696323, 1.32-foldrisk; rs12377632,1.30-fold risk) and three decreased-risk SNPs (rs3804099, 0.64-fold risk; rs3804100, 0.67-fold risk; rs11536889, 0.54-fold risk).

Previous PTB studies investigated the function of host genetic factors and immunoreactionin *M. tuberculosis* infection [11]. In humans, macrophages are the main host cells for the intracellular replication pathway of *M. tuberculosis*. Macrophages also serve as the antigen-presenting cells during the reactivation of lymphocytes, and they function as a vital killer of mycobacteria [12]. *In vivo* and *in vitro* studies show that *M. tuberculosis* infection causes apoptosis in monocytes and macrophages [13], and we previously found that apoptosis of these two cell types is a protective factor in human tuberculosis [14]. rs7696323 is located in the intron of the TLR2 gene located near 4q31.3 (precise position: 154605745) and rs11536889 is located in the 5’UTR of the TLR4 gene located near 9q33.1 (precise position: 120478131)has nothing to do with susceptibility of tuberculosis in Chinese Han children [15, 16]. The results showed significantly different, the reason may be genetic heterogeneity affects the identification of genetic factors in the disease, because there is no strict phenotypic boundaries between different genotypes in etiology. Secondly, because the age and nationality of the two groups are different, meanwhile the Children's immune system development is not fully awake so that can't fully open the immune defense [17],thus the results are not comparable. The SNP ofrs12377632 where is the promoter of the TLR4 gene located near 9q33.1 (precise position:120465144), Closely related to susceptibility to tuberculosis in our study. However, the correlation between the SNP and disease is rare [18–20]. Our study is the first to explore *TLR2* and *TLR4* polymorphisms and their association with PTB risk in a Tibetan Chinese population.

Our study was the first one to show that the variation of rs3804099 and rs3804100 in TLR2 influences the risk of tuberculosis (Figure 1). Compared with wild rs3804099 genotypes, the heterozygous mutation of type decreased TB risk, rs3804100 is the same way. The allele of rs3804099 and rs3804100located in the Coding exon, but the two cSNPs were considered to be synonymous. It has long been assumed the change of the encoding sequence caused by SNP does not affect the amino acid sequence of the translated protein. However, in recent studies found new evidence that the synonymous SNPs changes in exons can directly alter mRNA transcription and splicing, or Affect the expression of remaining exons within the genes, or affect the stability of mRNA via cis factors and modulate mRNA structure [21–24]. Our results show, the mutation of TLR2 gene may play an important protective role in the incidence of tuberculosis, which should be further explored.

Before the beginning of the study, many experimental results confirm TLR2 and TLR4 are implicated in the regulation of a variety of inflammatory and immune disorders [25–27]. Thus, we planned to evaluate the nine SNPs come from above two genes correlation in TB patients, but there are a few questions: (1) If the SNP associated with MTB in the some crowd, so does it apply to all crowd? (2) If certain mutations is significative, mought Tuberculosis occur? Although we confirmed some SNP can increase or decrease TB risk, the concrete mechanism is still to be further studied.

In conclusion, some limitations should be noted in the present study. Firstly, we studied only the Tibetan people, and sample content is relatively small. Secondly, the association between genetic polymorphism and different subtypes of Mycobacterium Tuberculosis, was not distinguished. To date, different lineages of M. tuberculosis strains cause the process of host detailed immune response is confirmed [28, 29]. This is very significant, different subtypes may match different SNPs [30]. But our study provides the first implied three the SNPs (rs7696323, rs3804099, rs3804100) come from TLR2 and two the SNPs (rs12377632, rs11536889) come from TLR4 indicated an association with MTB risk in the Tibetan Chinese population. But a basic molecular biologic are needed to confirm our findings in the future.

## MATERIALS AND METHODS

### Ethics statement

Our present study strictly observed the principles of the Declaration of Helsinki of the World Medical Association and was approved by the Ethics Committee of the Xi’an Jiaotong University Health Science center and the third Hospital of Tibet Autonomous Region. Informed consent forms were signed by all participants.

### Study participants

Between October 2012 and September 2013, we selected 971 individuals Tibetan PTB patients were recruited from the third Hospital of Tibet Autonomous Region in Lhasa, China, including 467 PTB patients and 504 healthy controls. All PTB patients were ethnic Tibetan and were newly diagnosed with consistent chest radiography and a positive sputum smear. Patients with human immunodeficiency virus (HIV), diabetes mellitus, or other tuberculosis diseases or who used immunosuppressive drugs were excluded. Individuals in the control group had no PTB history and no evidence of PTB in chest radiography or a positive sputum smear. We recruited subjects without consideration of age and gender.

### SNP selection

We selected nine *TLR2 and TLR4* SNPs with a minor allele frequency (MAF) above 5% in the HapMap Chinese Han Beijing population. We selected these SNPs on the basis of their allele frequencies, location, and disease relevance through public HapMap databases (http://www.Hapmap.org/index.html.en).

### Genotyping

Genomic DNA was extracted from peripheral blood samples using a genomic DNA purification kit (GoldMag, Xi’an, China). We used spectrometry (DU530 UV/VIS spectrophotometer, Beckman Instruments, Fullerton, CA) to measure the DNA concentration. The primers for amplification and extension reactions were designed with SequenomMassARRAY Assay Design 3.0 Software (Sequenom, San Diego, CA) [31]. We used SequenomMassARRAY RS1000 to perform the SNP genotyping with the agreement of the manufacturer [31], and we used Sequenom Typer 4.0 software for data management and analysis [31, 32].

### Statistical analysis

Microsoft Excel (Microsoft, Redmond, WA) and SPSS Statistics (version 17.0, SPSS, Chicago, IL) were used for statistical analyses. All *p*-values were two-tailed, and *p* ≤ 0.05 was considered to be statistically significant. SNP genotype frequencies in the case and control groups were calculated by Chi-square tests, and the Hardy-Weinberg equilibrium (HWE) was used to check the genotype frequency of the control group. Unconditional logistic regression analysis was used to examine the odds ratios (ORs) and 95% confidence intervals (CIs) in order to assess the association between SNPs and PTB [33]. Three models (dominant, recessive, log-additive) were used to test the association between SNPs and PTB [34]. Furthermore, Haploview (version 4.2, Broad Institute, Cambridge, MA) and SHEsis software were used for checking the linkage disequilibrium structure.

## Abbreviations

TLR: Toll-like receptor
PTB: Pulmonary tuberculosis
SNP: Single nucleotide polymorphism
OR: Odds ratio
CI: Confidence interval
MAF: minor allele frequency
HWE: Hardy-Weinberg Equilibrium
